# NADPH Oxidase-Mediated Activation of Neutral Sphingomyelinase Is Responsible for Diesel Particulate Extract-Induced Keratinocyte Apoptosis

**DOI:** 10.3390/ijms21031001

**Published:** 2020-02-03

**Authors:** Hyun-Seok Lee, Hye Yoon Park, Sung Pil Kwon, Bogyeong Kim, Yerin Lee, Seongeun Kim, Kyong-Oh Shin, Kyungho Park

**Affiliations:** 1 Research & Development Center, Chungdam CDC JNPharm LLC., Chuncheon 24232, Korea; 2 Biological and Genetic Resources Assessment Division, National Institute of Biological Resources, Incheon 22689, Korea; 3 Department of Food Science & Nutrition, and Convergence Program of Material Science for Medicine and Pharmaceutics, Hallym University, Chuncheon 24252, Korea; 4 Korean Institute of Nutrition, Hallym University, Chuncheon 24252, Korea

**Keywords:** diesel particulate extract, keratinocyte apoptosis, NADPH oxidase, ceramide, neutral sphingomyelinase

## Abstract

Human epidermis is positioned at the interface with the external environment, protecting our bodies against external challenges, including air pollutants. Emerging evidence suggests that diesel particulate extract (DPE), a major component of air pollution, leads to impairment of diverse cellular functions in keratinocytes (KC). In this study, we investigated the cellular mechanism underlying DPE-induced KC apoptosis. We first addressed cell death occurring in KC exposed to DPE, paralleled by increased activation of NADPH oxidases (NOXs) and subsequent ROS generation. Blockade of NOX activation with a specific inhibitor attenuated the expected DPE-induced KC apoptosis. In contrast, pre-treatment with a specific inhibitor of reactive oxygen species (ROS) generation did not reverse DPE/NOX-mediated increase in KC apoptosis. We next noted that NOX-mediated KC apoptosis is mainly attributable to neutral sphingomyelinase (SMase)-mediated stimulation of ceramides, which is a well-known pro-apoptotic lipid. Moreover, we found that inhibition of NOX activation significantly attenuated DPE-mediated increase in the ratio of ceramide to its key metabolite sphingosine-1-phosphate (S1P), an important determinant of cell fate. Together, these results suggest that activation of neutral SMase serves as a key downstream signal for the DPE/NOX activation-mediated alteration in ceramide and S1P productions, and subsequent KC apoptosis.

## 1. Introduction

The epidermis is the outermost layer of skin, and protects our bodies against external perturbants, e.g., UV irradiation, microbial pathogen, and air pollution, which potentially threaten the integrity of the epidermal permeability barrier [1]. Recent evidence reveals that air pollutants lead to impairment of epidermal barrier function; and diesel particulate extract (DPE) is one of the major components of air pollution, as it is the most abundant combustion-derived particle in traffic emission [2,3]. DPE consists of a heterogeneous mixture of volatile components, including aldehydes, benzene, butadiene, polyaromatic hydrocarbons (PAHs) and their derivatives [4]. Prior studies have demonstrated that cellular mechanisms underlying DPE-mediated detrimental effects are mainly initiated by the generation of oxidative stress [5]. Specifically, PAHs such as benzopyrene are known to be major contributors to the development of diverse skin diseases, e.g., contact hypersensitivity and dermatitis, or impairment of cellular functions of epidermal keratinocytes (KC), i.e., increased KC apoptosis [6,7], through oxidative stress-mediated mechanisms.

Nicotinamide adenine dinucleotide phosphate (NADPH) oxidases (NOXs) are proteins that transfer electrons across biological membranes and are responsible for generation of reactive oxygen species (ROS), including both superoxide and hydrogen peroxide, leading to induction of oxidative stress [8,9]. Air pollutants, including DPE, have been shown to activate NOXs and ROS production, leading to cellular apoptosis [10,11]. Subsequently, NOX activation-mediated increase in ROS activates a key group of ceramide-generating enzymes, the sphingomyelinases (SMases), resulting in stimulation of ceramide production [12]. Although ceramide is an effective structural component for the formation of the epidermal permeability barrier, emerging evidence demonstrates that excessive accumulation of cellular ceramides is involved in the induction of apoptosis in multiple tissues/cell types, including KC, via previously identified downstream mechanisms, i.e., bax/caspase caspase-dependent pathway [13]; stress-activated protein kinase (SAPK)/c-jun kinase (JNK) cascade-dependent pathway [14]; and imbalanced phosphoinositide 3-kinase (PI3K)-Akt/PKB (protein kinase B) pathway [15]. But, the detailed mechanism(s) for the DPE-mediated KC apoptosis is (are) not clearly understood. 

In the present study, we investigated whether NOX-mediated downstream mediators; e.g., ROS and/or ceramide, could lead to apoptotic cell death in KC exposed to DPE. Our results demonstrate that DPE-mediated activation of NOXs activates neutral SMase, in turn stimulating cellular ceramide production that results in KC apoptosis. Contrary to previous results, interestingly, our findings revealed that DPE-mediated stimulation of ceramide is attributed to a ROS-independent mechanism. As such, the insights from these studies could lead to the development of novel mechanism-based therapies for diseases associated with KC apoptosis.

## 2. Results

### 2.1. Diesel Particulate Extract (DPE) Induces Keratinocytes (KC) Apoptosis, Paralleled by Increased NAPDH Oxidation and Reactive Oxygen Species (ROS) Generation 

**Figure 1:**
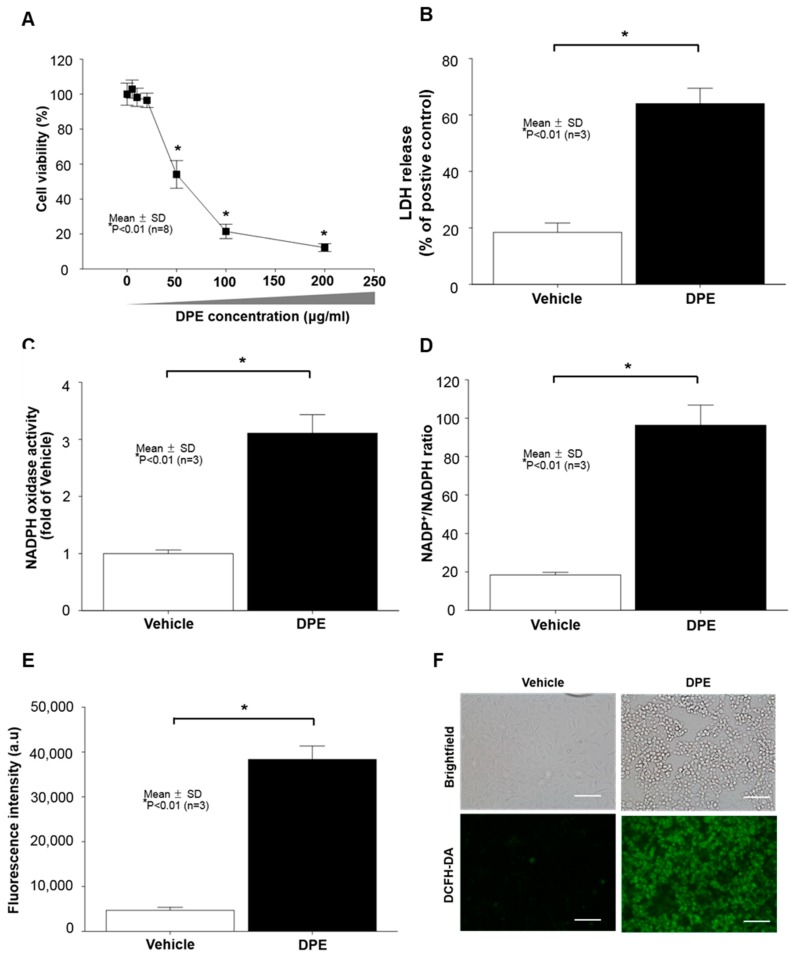
Diesel particulate extract (DPE) induces keratinocyte (KC) apoptosis, paralleled by increased NADPH oxidase activity and reactive oxygen species (ROS) production. Human HaCaT KC were treated with DPE (100 μg/mL or as indicated) for 24 hrs. Cell viability was measured by a CCK-8 assay kit (**A**). Cell cytotoxicity was further determined by an LDH assay and results were expressed as LDH release compared to the positive control consisting of 0.1% SDS (yielding 100% LDH release) (**B**). In order to measure the levels of NADPH oxidation, cell membrane fractions were obtained as described in Materials and Methods. Activity of NAPDH oxidases was determined by either lucigenin chemiluminescence assay using *N*,*N*′-Dimethyl-9,9′-biacridinium dinitrate (**C**) or measuring the ratio of NADP+ to NADPH with LC-ESI-MS/MS (API 3200 QTRAP mass, AB/SCIEX) by multiple reaction monitoring mode (MRM) (**D**). Intercellular ROS production was determined by either a fluorospectrophotometer (**E**) or fluorescence microscopy (**F**) with the oxidant-sensing probe 2′,7′-dichlorodihydrofluorescein diacetate (DCFH-DA). All values are mean ± SD (*n* = 3). Statistical significance was calculated using the unpaired Student’s *t*-test, and significance was defined as *p* < 0.01 vs. vehicle control (or untreated control). Bar = 500 μm.

Diesel particulate extract (DPE) has been shown to induce cell death in different cell types, including keratinocytes (KC) [4]. We first determined what concentration of DPE alters KC growth in our experimental conditions. WST assay revealed a significant dose-dependent decrease in cell viability in human KC after incubation with DPE (1 to 200 μg/mL) (Figure 1A). Because 78.5% of cells died when DPE concentration reached 100 μg/mL (Figure 1A), we used DPE at concentrations of 100 μg/mL in subsequent studies. Our lactate dehydrogenase (LDH) release analysis further confirmed that 100 μg/mL of DPE significantly attenuates cell viability (Figure 1B). Prior studies revealed that external stresses, such as UV irradiation, air pollution, and chemicals, trigger activation of NADPH oxidases (NOXs) and subsequent reactive oxygen species (ROS) generation. Our LC-MS/MS analysis revealed that DPE exposure stimulates both NOX activity (Figure 1C,D) and ROS production (Figure 1E,F). These results suggest that DPE-induced KC apoptosis is paralleled by stimulated NOX activation and ROS production.

### 2.2. DPE Induces KC Apoptosis through NOX Activation, but Not ROS-Dependent Mechanism

**Figure 2:**
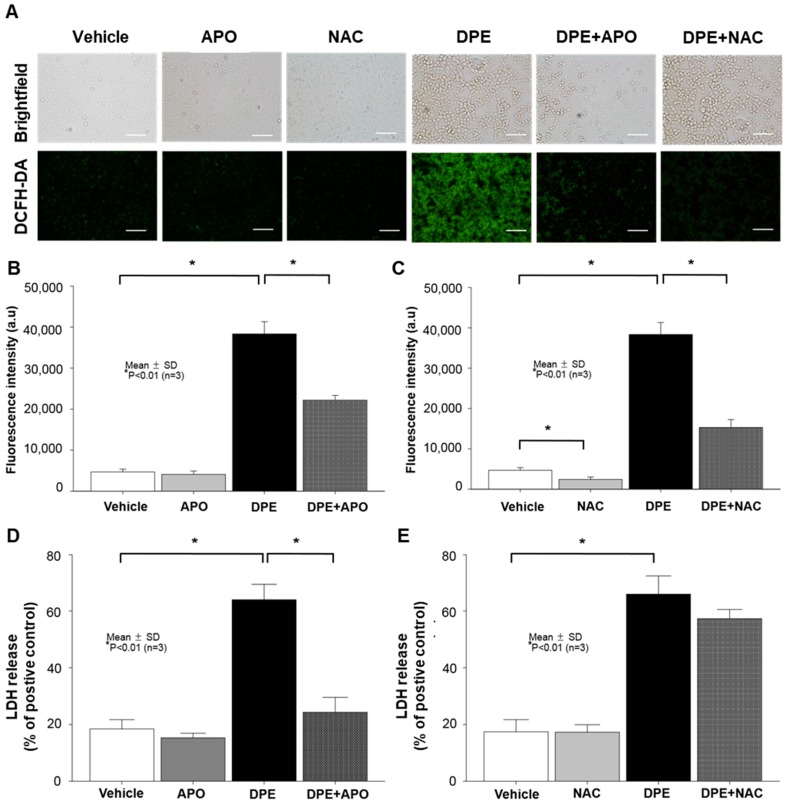
DPE-induced KC apoptosis through activation of NADPH oxidases (NOXs), but not through ROS-dependent pathways. Human KC pre-treated with or without NOX (Apocynin [APO], 100 μM) or ROS generation (N-Acetylcysteine [NAC], 1 mM) inhibitors for 30 mins were incubated with DPE (100 μg/mL) for 24 hrs. Intercellular ROS production was determined by either a fluorescence microscopy (**A**) or fluorospectrophotometer (**B**,**C**) with the oxidant-sensing probe 2′,7′-dichlorodihydrofluorescein diacetate (DCFH-DA). Cell cytotoxicity was measured by an LDH assay and results were expressed as LDH release compared to the positive control consisting of 0.1% SDS (yielding 100% LDH release) (**D**,**E**). All values are mean ± SD (*n* = 3). Statistical significance was calculated using the unpaired Student’s *t*-test, and significance was defined as *p* < 0.01 vs. vehicle control (or untreated control). Bar = 500 μm.

To further ascertain whether NOX activation-induced stimulation of ROS production is responsible for the DPE-mediated increase in KC apoptosis, we blocked either NOX activation or ROS generation using appropriate pharmacological inhibitors, Apocynin (APO) or N-Acetylcysteine (NAC), respectively. Again, intercellular stimulation of ROS became evident in KC following DPE exposure (Figure 2A–C). However, pretreatment of DPE-treated KC with APO or NAC significantly attenuated the expected increase in ROS generation (Figure 2A–C). In addition, DPE increases LDH activity (Figure 2D,E), whereas, inhibition of NOX activation with APO treatment significantly attenuated the DPE-mediated increase in LDH activity (Figure 2D). However, blockade of ROS generation by NAC did not diminish the DPE-mediated increase in LDH activity (note: a modest decrease in LDH release was found, compared to that of DPE alone, but there was no statistically significant difference) (Figure 2F). These results suggest that DPE induces KC apoptosis through activation of NOXs, but ROS, a downstream mediator of NOX activation, is not likely involved in DPE-induced KC apoptosis.

### 2.3. DPE-Induced Activation of NAPDH Oxidation is Responsible for Increased Overall Ceramide Production in Human KC

**Figure 3:**
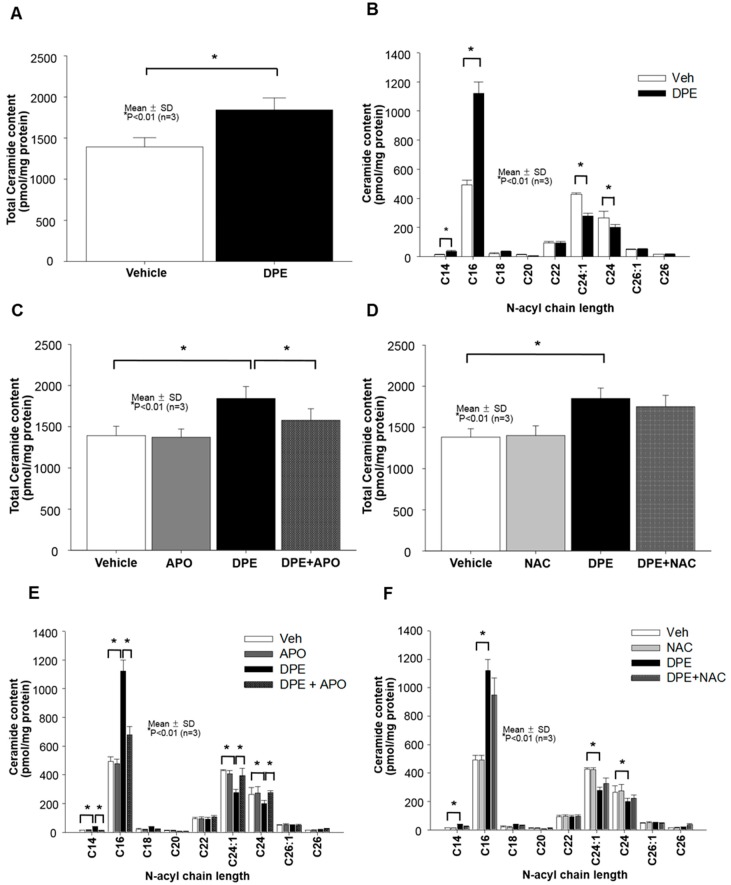
DPE-mediated activation of NOXs accounts for stimulated production of total ceramides. Human KC pre-treated with or without NOX (Apocynin [APO], 100 μM) or ROS generation (N-Acetylcysteine [NAC], 1 mM) inhibitors for 30 mins were incubated with DPE (100 μg/mL) for 24 hrs. Total ceramides (**A**,**C**,**D**) and ceramides containing different carbon chain lengths of fatty acid (**B**,**E**,**F**) were assessed by LC-ESI-MS/MS. All values are mean ± SD (*n* = 3). Statistical significance was calculated using the unpaired Student’s *t*-test, and significance was defined as *p* < 0.01 vs. vehicle control (or untreated control).

Prior studies have shown that NOX activation-mediated increase in ROS could stimulate cellular levels of ceramide (a well-known pro-apoptotic lipid) [12]. Thus, we next assessed whether DPE alters ceramide production in KC and found a significant increase in production of total ceramide in cells exposed to DPE (Figure 3A,C,D). While all ceramides are composed of sphingosine and fatty acids (FAs), differences in carbon chain lengths of FAs in ceramides have been reported to affect distinct cellular functions in skin, including apoptosis; i.e., ceramides carrying short chain fatty acids (< C20) and relatively long chain FAs (>C22) are pro-apoptotic or anti-apoptotic, respectively [16]. Therefore, we further investigated the carbon chain lengths of ceramide FAs. Our lipid analysis revealed that DPE treatment significantly increases short-chain ceramide levels (C14-C20), while in contrast, levels of ceramide containing long-chain FAs (C24:0 and C24:1) decreased in cells exposed to DPE (Figure 3B). The DPE-induced changes in ceramide species were reversed back to basal levels by inhibition of NOX activation (Figure 3C,E), but blockade of ROS generation did not alter DPE-induced changes in ceramide species (Figure 3D,F). These results indicate that NOX activation accounts for DPE-mediated stimulation of ceramide. In particular, ceramides containing short chain FAs were increased, and conversely, long chain FA-containing ceramides were diminished in KC exposed to DPE. 

### 2.4. Neutral Sphingomyelinase (SMase) Is Required for DPE/NOX Activation-Mediated Increase in Ceramide Production and Subsequent KC Apoptosis

**Figure 4:**
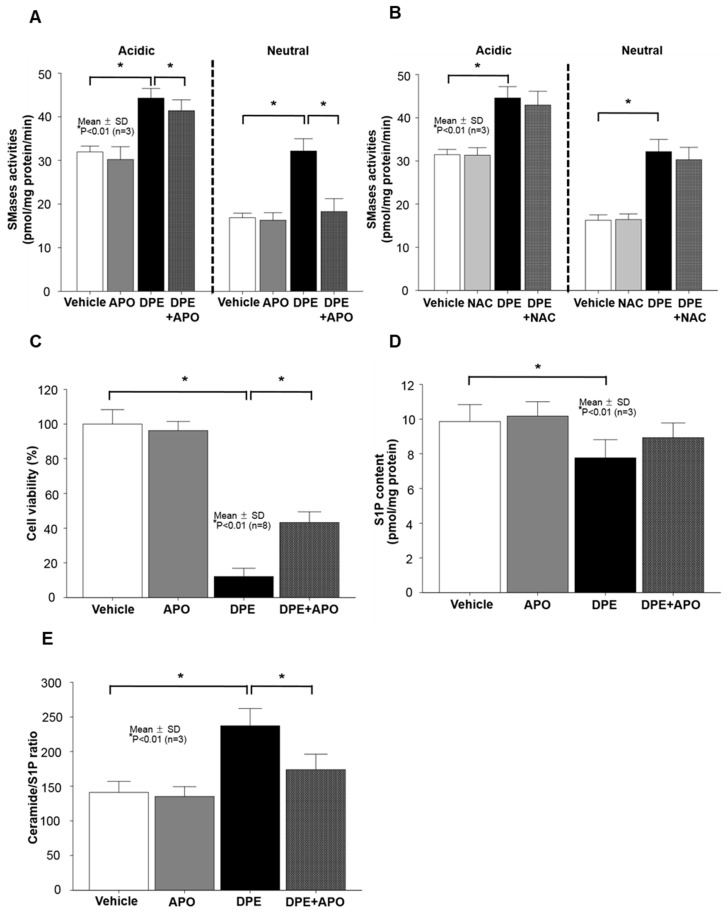
DPE-mediated activation of neutral sphingomyelinase (SMase) is required for NOX activation-induced increase in ceramide and subsequent KC apoptosis. Human KC pre-treated with or without NOX (Apocynin [APO], 100 μM), ROS generation (N-Acetylcysteine [NAC], 1 mM), or GW4869 (neutral SMase inhibitor, 10 μM) for 30 mins were incubated with DPE (100 μg/mL) for 24 hrs. Activities of acidic and neutral SMases (**A**,**B**), and contents of ceramide and sphingosine-1-phosphate (**D**,**E**) were assessed by LC-ESI-MS/MS. Cell viability or cytotoxicity was measured by either WST-1 assay (**C**). All values are mean ± SD (*n* = 3). Statistical significance was calculated using the unpaired Student’s *t*-test, and significance was defined as *p* < 0.01 vs. vehicle control (or untreated control).

Prior studies demonstrated that cell membrane ceramides are mainly produced from sphingomyelin, the major lipids in the lipid bilayer, via the hydrolysis pathway by either neutral sphingomyelinase (SMase) or/and acidic SMase actions [17]. Therefore, we next addressed both SMase activities in KC exposed to DPE. Enzyme activity assay revealed an increase in activities of both SMase isoforms in cells following DPE treatment (Figure 4A,B). Next, we determined whether NOX activation is responsible for the DPE-mediated activation of both SMases. Treatment with APO, an inhibitor of NOX activation, significantly diminished the DPE-induced activation of neutral SMase, whereas the DPE-mediated increase in activity of acidic SMase remained elevated in cells treated with APO (Figure 4A). We next assessed the involvement of ROS in the DPE-mediated increase in ceramide production. As shown in Fig. 4b, blockade of ROS generation by NAC did not reverse the DPE-induced stimulation of ceramide production. To ensure the impact of neutral SMase on DPE-induced KC apoptosis, cells were co-treated with DPE and GW4869, an inhibitor of neutral SMase, and found that suppression of neutral SMase activity significantly attenuates DPE-induced cell death (Figure 4C). These results indicated that activation of neutral SMase, but not acidic SMase, serves as a key downstream signal for DPE/NOX activation-mediated stimulation of ceramide production and subsequent KC apoptosis.

Moreover, while ceramides have a prominent role in the regulation of apoptosis, one key metabolite, sphingosine-1-phosphate (S1P), is largely recognized as an anti-apoptotic lipid [18]. Thus, we further assessed whether DPE-mediated activation of NOXs alters cellular levels of S1P. A decrease in S1P levels was found in cells after exposure to DPE (Figure 4D), but DPE-induced decrease in S1P did not change in cells treated with APO (although a modest increase in S1P was noted, there was no statistically significant difference) (Figure 4D). Interestingly, however, an inhibition of NOX activation by APO treatment significantly reduced the DPE-mediated increase in the ceramide to S1P ratio (Figure 4E). 

## 3. Discussion

Ceramides are mainly generated through two of the following pathways [19]: (i) de novo pathway, initiated by serine palmitoyltransferase; (ii) sphingomyelin (SM) hydrolysis pathway, initiated by either neutral sphingomyelinase (SMase) and/or acidic SMase. In particular, prior studies demonstrated that neutral SMase is the most abundant isoform within the plasma membrane of KC and is the prevailing factor in sphingomyelin-ceramide homeostasis regulation [17]. While the former pathway accounts for cell survival or normal metabolic functions [20], the latter is activated in response to various apoptotic stimuli, such as chemical toxicants, pathogen infection, UV irradiation, inflammation, or hypoxia, leading to induction of the apoptosis pathway [15]. Recently, high levels of air pollutants, including diesel particulate extracts (DPE), has been classified as a potential contributor to activation of membrane-bound oxidases, NADPH oxidases (NOXs), which in turn stimulates productions of their downstream mediators, ceramides and/or reactive oxygen species (ROS), resulting in induction of apoptosis [19]. Pertinently, our present study suggests that DPE-mediated increase in overall NOX oxidation activates neutral SMase within the plasma membrane of KC, leading to stimulation of ceramide generation, which in turn induces apoptosis. But, further studies are still needed to identify which specific isoform(s) of NOXs is (are) responsible for DPE-induced activation of neutral SMase. Moreover, our studies further revealed that DPE-mediated NOX activation selectively increases cellular levels of ceramides carrying relatively short chain fatty acids (< C20), which is consistent with prior studies showing that short chain ceramides are recognized as apoptotic lipids [16]. In contrast to increased ceramide contents, NOX activation-mediated increase in production of ROS did not account for DPE exposure-mediated induction of KC apoptosis. Compared to other tissues, the skin continuously faces a hostile external environment, and it exhibits a higher resistance to elevated ROS-mediated increase in oxidative stress due to an increased expression/activation of nuclear erythroid 2-related factor (Nrf2), a key player in the antioxidant defense mechanism [21]. Specifically, elevated ROS generation leads to an increase in expression and/or activity of Nrf2, providing a redox homeostasis through a dynamic equilibrium between ROS production and scavenging [21]. In addition, oxidative stress/ROS production is required for normal skin functions; i.e., keratinocyte differentiation, an essential process in the formation of the skin permeability barrier. Toxic levels or prolonged exposure to ROS induce an imbalance in the cellular antioxidant defense mechanism, causing several harmful consequences, including apoptosis [22]. Accordingly, the present study suggests that the levels of ROS produced by our experimental conditions; e.g., concentration or incubation period of DPE, are not sufficient to induce KC apoptosis. Moreover, we further speculate that increased expression and/or activity of antioxidant genes, such as Nrf2, might have occurred under our experimental conditions, and would be enough to overcome cell death caused by DPE-induced production of ROS. 

Ceramide’s key metabolite (sphingosine-1-phosphate [S1P]) promotes cellular division as opposed to apoptosis [18]. In fact, ceramides are hydrolyzed by ceramidases to sphingosine, which is phosphorylated by sphingosine kinases into S1P [19]. Our prior studies demonstrated that metabolic conversions of ceramide to S1P by the actions of appropriate metabolic enzymes protect KC against UVB irradiation-induced, ceramide-mediated cell death [23]. In the present study, although a blockade of NOX activation by APO treatment did not reverse the DEP-mediated decrease in S1P levels, APO-mediated increase in cell survival is in part attributed to a decreased ceramide to S1P ratio. These results further support prior findings that cell fate could be determined by the balance between ceramide and S1P [24].

**Figure 5:**
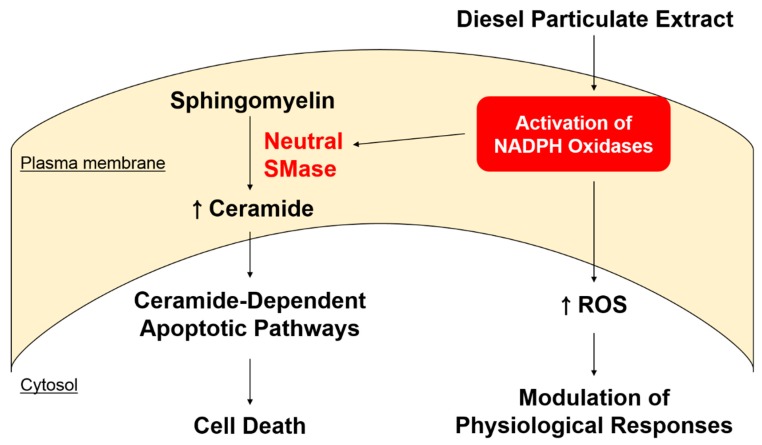
Proposed mechanism for DPE-mediated induction of KC apoptosis. DPE-mediated activation of NOXs triggers activation of neutral SMase, in turn inducing KC apoptosis through increased ceramide production, rather than through a ROS-dependent mechanism.

In summary, our present studies illuminate how DPE-mediated NOX activation induces KC apoptosis; i.e., DPE-mediated activation of NOXs then activates neutral SMase, leading to induction of KC apoptosis through increased ceramide production, but not through a ROS-dependent mechanism (see Figure 5). Because oxidative stress-induced cell apoptosis is implicated in not only skin disorders such as aging, inflammation, but also in ischemia/reperfusion injury, and neurodegenerative diseases such as Alzheimer’s and Parkinson’s disease, our studies further suggest that pharmacological modulation of oxidative stress by regulating the levels of NOX and neutral SMase activations could represent a therapeutic approach for such diseases.

## 4. Materials and Methods

### 4.1. Reagents

Diesel particulate extract (DPE) used in the present study is Standard Reference Material 1975 (SRM 1975), purchased from the National Institute of Standards and Technology (NIST) (Gaithersburg, MD, USA). Apocynin, N-acetylcysteine, nicotineamide, nicotinamide adenine dinucleotide phosphate (NADPH), NADP, and 2′,7′-dichlorofluorescin diacetate were obtained from Sigma-Aldrich (St. Louis, MO, USA). C17-sphingosine-1-phosphate (C17-S1P), S1P (d18:1), sphingosine, ceramides (fatty acid lengths C12, C16, C18, C22, C24, and C24:1), C17-ceramide (d17:1/C18:0), and C12-sphingomyeline were obtained from Avanti Polar Lipids (Alabaster, AL, USA). Organic solvents for sphingolipid extraction or LC-MS/MS analysis were purchased from Merck (Darmstadt, Germany). Unless otherwise stated, all other chemicals were obtained from Sigma-Aldrich.

### 4.2. DPE Preparation

DPE corresponds to a dichloromethane extract of filter-collected combustion particulate matter from operating forklifts with diesel engines (for further details see [25]). Dichloromethane was evaporated under nitrogen gas, and the final residue was dissolved in stock solvent (dimethyl sulfoxide [DMSO]: fetal bovine serum [FBS], 9:1, *v*/*v*; stock concnetration of DPE= 100 mg/mL). Prior to cell exposure, 1 ml of DPE stock solution was further diluted in 9 ml of cell culture medium (concentration of DPE: 10 mg/mL), followed by sonication for 30 mins at 37 °C.

### 4.3. Cell Culture

Immortalized, non-transformed (HaCaT) human keratinocytes (KC), derived from human epidermis (a gift from N. Fusenig, Heidelberg, Germany), were grown as described previously [26]. Briefly, HaCaT KC were maintained in Dulbecco’s modified Eagle’s medium (DMEM) containing 10% fetal calf serum (FCS) and 1% penicillin/streptomycin (P/S). Similar to our prior studies [27], culture medium was switched to serum-free, antibiotics-free KC growth medium (154CF, Thermo Fisher Scientific, Carlsbad, CA, USA) containing 0.07 mM calcium chloride and growth supplements (Thermo Fisher Scientific, Carlsbad, CA, USA) 1 day prior to DPE treatment.

### 4.4. Cell Viability 

Cell viability or cytotoxicity was measured by the water-soluble tetrazolium salt (WST) method using the cell counting kit-8 (CCK-8) assay kit (Dojindo, Japan) in accordance with the manufacturer’s instruction. Cells (3 × 104 cells/well, 96-well plate) pre-treated with or without NOX (Apocynin [APO], 100 μM) or ROS generation (N-Acetylcysteine [NAC], 1 mM) inhibitors for 30 mins were incubated with DPE (100 μg/mL) for 24 hrs. Ten μL of CCK-8 solution was then added into each well and the plate was incubated for 1 hr prior to reading absorbance in a microplate reader (Molecular devices M2e, Molecular Devices, Sunnyvale, CA, USA) at 450 nm. The relative cell viability was calculated as the percentage of vehicle-treated cells.

### 4.5. Lactate Dehydrogenase Assay

DPE-induced cytotoxicity was further determined by measuring lactic dehydrogenase (LDH) release using an in vitro LDH based toxicology assay kit Tox-7 (Sigma-Aldrich, St. Louis, MO, USA). Briefly, HaCaT KC pretreated with APO (100 μM) or NAC (1 mM) were incubated with DPE (100 μg/mL) or 0.1% sodium dodecyl sulfate (SDS) as a positive control for 24 hrs. Solutions were prepared following the manufacturer’s instructions and the absorbance of the final assay solution was measured at 490 nm using a microplate reader (Molecular devices M2e, Molecular Devices, Sunnyvale, CA, USA). The relative cytotoxicity (% of positive control) was expressed as LDH release compared to the positive control consisting of 0.1% SDS (yielding 100% LDH release).

### 4.6. Activity of NADPH Oxidases

To measure activity of NADPH oxidase, cell membrane fractions were obtained as described previously [28]. NADPH oxidase activity was determined by the lucigenin chemiluminescence assay kit using *N*,*N*′-Dimethyl-9,9′-biacridinium dinitrate (Sigma-Aldrich, St. Louis, MO, USA) and 1 μM NADPH (Sigma-Aldrich, St. Louis, MO, USA), in accordance with the manufacturer’s instruction.

In addition, we further assessed the activity of NADPH oxidases by measuring the ratio of NADP+ to NADPH with LC-ESI-MS/MS (API 3200 QTRAP mass, AB/SCIEX, Framingham, MA) by multiple reaction monitoring (MRM) mode, as described previously with minor modifications. Briefly, to extract NADPH and NADP+ from cell membrane fractions, proteins (10 μg) extracted from cell membrane fractions were incubated in a reaction buffer (10 mM nicotinamide, 20 mM NaHCO3, 100 mM Na2CO3, 0.05% Triton X-100, and 1% DTAB [D8638; Sigma-Aldrich, St. Louis, MO, USA] containing 200 μM of NADPH) for 30 min at 37 °C. The reaction was terminated by adding cold acetonitrile (750 μL). Extracted NADPH and NADP+ were analyzed by LC-ESI-MS/MS (API 3200 QTRAP mass, AB/SCIEX, Framingham, MA) by MRM mode. The metabolites were separated on a Phenomenex Kinetex C18 column (2.1 × 50 mm, 2.6 μm) with an injection volume (5 μL) and a flow rate of 0.6 ml/min using 10 mM ammonium acetate for mobile phase A and methanol for mobile phase B. The gradient was as follows: 0 min, 0% B; 1.5 min, 30% B; 1.51 min, 95% B; 1.8 min, 95% B; 1.81 min, 0% B, 3 min, 0% B. The NADPH and NADP+ MS/MS transitions (m/z) were 744→508 for NADP+ and 746→729 for NADPH.

### 4.7. 2′,7′-Dichlorodihydrofluorescein Diacetate-Based Detection of Cellular ROS

To detect production of cellular reactive oxygen species (ROS), including superoxide (O_2_^-^) and hydrogen peroxide (H_2_O_2_), the oxidant-sensing probe 2′,7′-dichlorodihydrofluorescein diacetate (DCFH-DA) was used as described previously [29]. DCFH-DA used in the present study was obtained from abcam (Cambridge, MA, USA) and the assay was performed in accordance with the manufacturer’s instruction. ROS production was analyzed using either a fluorescence microscopy (Eclipse Ti-U; Nikon Corporation, Tokyo, Japan) or fluorospectrophotometer (Molecular devices M2e, Molecular Devices, Sunnyvale, CA, USA) with 485 nm of excitation and 520 nm of emission filters and was expressed as a fluorescence intensity (a.u.).

### 4.8. Measurement of Ceramide and Sphingosine-1-Phosphate (S1P) 

To assess the levels of cellular sphingolipids, such as ceramides and S1P, human KC pre-treated with or without NOX (Apocynin [APO], 100 μM) or ROS generation (N-Acetylcysteine [NAC], 1 mM) inhibitors for 30 mins were incubated with DPE (100 μg/mL) for 24 hrs, followed by extraction of sphingolipids as we reported previously [30,31]. The extracted lipids dried using a vacuum system (Vision, Seoul, Korea) were re-dissolved in methanol and analyzed by LC-ESI-MS/MS (API 3200 QTRAP mass, AB/SCIEX, Framingham, MA, USA) by selective ion monitoring mode. The ceramide MS/MS transitions (m/z) were 510→264 for C14-ceramide, 538→264 for C16-ceramide, 552→264 for C17-ceramide, 566→264 for C18-ceramide, 594→264 for C20-ceramide, 648→264 for C24:1-ceramide, 650→264 for C24-ceramide, 676→264 for C26:1-ceramide, and 678→264 for C26-ceramide, respectively. The S1P MS/MS transitions (m/z) were 366→250 for C17 S1P as an internal standard and 380→264 for C18 sphingosine 1-phosphate, respectively. Data were acquired using Analyst 1.5.1 software (Applied Biosystems, Foster City, CA). Sphingolipid levels are expressed as pmol per mg protein.

### 4.9. Enzyme Activity Assay for Sphingomyelinases (SMases)

Activities of acidic or neutral SMases were assessed, as described previously [32]. Briefly, cells suspended in appropriate SMase assay buffers (acdic SMase buffer: 250 mM sodium-acetate, 0.2% Triton X-100, pH 4.5 or neutral SMase buffer: 20 mM HEPES, 0.2% Triton X-100, pH 7.4) were incubated with 5 nmol of C12-sphingomyeline for 20 min at 37 °C. The reaction was stopped by the addition of CHCl3: CH3OH (2:1, *v*/*v*), and the organic phases were removed under N2 gas. The residues then were resuspended in MeOH and applied onto LC-MS/MS system. The activities of both SMases are expressed as pmol (C12-ceramide) per mg protein per min.

### 4.10. Statistical Analyses

Results were expressed as the mean ± standard deviation (SD). Statistical analyses were performed using the Prism Version 6.0 Software (GraphPad Software, San Diego, CA, USA). Significance between groups was determined by unpaired Student t test. The P values were set at <0.01.

## Abbreviations

APO	Apocynin
DCFH-DA	2′,7′-dichlorodihydrofluorescein diacetate
DPE	Diesel particulate extract
FA	Fatty acid
JNK	c-jun kinase
KC	Keratinocytes
LDH	Lactate dehydrogenase
MRM	Multiple reaction monitoring mode
NAC	N-Acetylcysteine
NADPH	Nicotinamide adenine dinucleotide phosphate
NOX	NADPH oxidase
PAH	Polyaromatic hydrocarbon
PI3K/PKB	Phosphoinositide 3-kinase/ Protein kinase B
ROS	Reactive oxygen species
SAPK	Stress-activated protein kinase
SMase	Sphingomyelinase
S1P	Sphingosine-1-phosphate
WST	Water-soluble tetrazolium salt
